# Experimental Study of Fire Hazards of Thermal-Insulation Material in Diesel Locomotive: Aluminum-Polyurethane

**DOI:** 10.3390/ma9030168

**Published:** 2016-03-05

**Authors:** Taolin Zhang, Xiaodong Zhou, Lizhong Yang

**Affiliations:** 1 State Key Laboratory of Fire Science, University of Science and Technology of China, 96 Jinzhai Road, Hefei 230026, China; ztlfire@mail.ustc.edu.cn (T.Z.); zxd@ustc.edu.cn (X.Z.); 2 Collaborative Innovation Center for Urban Public Safety, 96 Jinzhai Road, Hefei 230026, China

**Keywords:** insulation material, diesel locomotive, heat flux, toxic gas concentration, hazard index

## Abstract

This work investigated experimentally and theoretically the fire hazards of thermal-insulation materials used in diesel locomotives under different radiation heat fluxes. Based on the experimental results, the critical heat flux for ignition was determined to be 6.15 kW/m^2^ and 16.39 kW/m^2^ for pure polyurethane and aluminum-polyurethane respectively. A theoretical model was established for both to predict the fire behaviors under different circumstances. The fire behavior of the materials was evaluated based on the flashover and the total heat release rate (HRR). The fire hazards levels were classified based on different experimental results. It was found that the fire resistance performance of aluminum-polyurethane is much better than that of pure-polyurethane under various external heat fluxes. The concentration of toxic pyrolysis volatiles generated from aluminum-polyurethane materials is much higher than that of pure polyurethane materials, especially when the heat flux is below 50 kW/m^2^. The hazard index *HI* during peak width time was proposed based on the comprehensive impact of time and concentrations. The predicted *HI* in this model coincides with the existed N-gas and FED models which are generally used to evaluate the fire gas hazard in previous researches. The integrated model named HNF was proposed as well to estimate the fire hazards of materials by interpolation and weighted average calculation.

## 1. Introduction

Thermal-insulation materials are widely used in the diesel locomotive for reducing energy consumption. Organic insulation materials, such as polyurethane, characterized by high intensity, low density, corrosion resistance and low price, were used extensively in constructions. However, numerous diesel locomotive fires have caused severe casualties and property loss recently, which attracted the public attention of fire safety researchers. The combustion of those organic macromolecule materials accompanied by high temperature, huge HRR and fast fire growth produces a great amount of smoke and toxic gases.

The fire hazards of materials have long been the concern of researchers. Due to many variable factors in the burning process, the evaluation of fire hazards of materials is complicated [1]. The complex process of the pyrolysis and ignition of combustible solid exposed to radiation is a combination of heat and mass transfer in solid-gas phase, chemical reactions and gas flow. Due to the importance and complication of the process in fires, the related research has been conducted since the 40s of last century [2]. Since the development of a cone calorimeter for testing the combustion characteristics of materials by NIST in 1982, researchers have done a lot of experimental and theoretical studies for the pyrolysis and ignition of solid combustibles, which considered the physical and chemical processes and variable changes. The pyrolysis and ignition models have been proposed based on the related assumptions [3]. Janssens [4], Atreya [5], Babrauskas [6], Quintiere [7] and Di Blasi *et al.* [8] conducted various studies to predict the early stage of fires based on the comparison between experimental results and the modified models. Delichatsios [9,10,11] and other researchers studied the relationship between the ignition time and external heat flux with the existence of ignition source when the sample was horizontally positioned and the heat flux varies between 20–100 kW/m^2^. Kashiwagi [12], Atreya [13], Shields [14], Merryweather [15] and Tsai *et al.* [16] studied the effects of radiant heat directions on the ignition time, critical heat flux, HRR and fire spread. Biteau [17], Valencia [18], Chow [19], Hull [20], Luche [21] and Stec *et al.* [22] studied the fire hazards and combustion behavior of solid materials using the cone calorimeter.

Most casualties in fires are not caused by burns but by the inhalation of toxic gases in smoke, especially the carbon monoxide (CO), which is thought to be the major cause of deaths in fires. Based on the researches for the mechanism of biological effect, the index to evaluate fire hazards of the material has been proposed, among which, LCB_50B_ (the 50% of exposure death in a given time) and ICB_50B_ (the 50% of exposure function loss in a given time) are most broadly used. To evaluate the fire hazards of the major toxic gases in fires, previous researchers have progressed N-gas model. FED model has integrated the LC50 criterion and N-gas model, which takes the accumulation effect of time into consideration. A linear relationship is hypothesized between the fire hazard and different species [23,24,25,26,27,28].

The polyurethane and other thermosetting materials have been broadly used in the cooling room of diesel locomotive for their unique thermal resistance quality. A comprehensive study of the characteristics of fire hazards of organic insulation materials, especially the aluminum-polyurethane, in different radiation heat fluxes is of vital importance in terms of safety issue. In this paper, a series of experiments were conducted to study the performance of the structural and ornamental thermal-insulation materials during pyrolysis and ignition process. In addition, an integrated model was developed to predict these behaviors, and the model was verified to be pretty good through the comparison between the model and the experimental results.

## 2. Experimental Section

### 2.1. Materials

**Figure 1:**
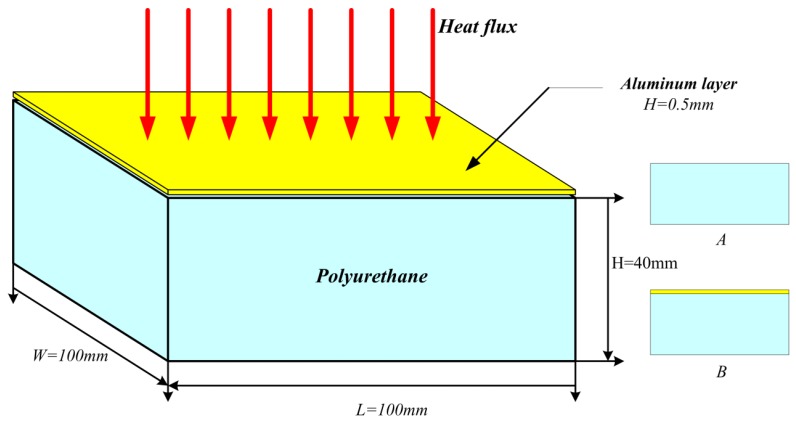
The structure of aluminum-polyurethane and pure polyurethane.

As shown in Figure 1, aluminum-polyurethane plates were used in the experiments. The dimensions of the polyurethane were 100 mm × 100 mm × 40 mm (length × width × height). The density is 30 kg/m^3^ and bending strength is 0.196 MPa. A 0.5 mm aluminum sheet was attached on top of the polyurethane which thermal conductivity is 0.039 W/(m.K).

The experimental materials, denoted by A and B, are pure polyurethane, and aluminum-polyurethane plate, respectively. Before the experiments, all the samples have been carved up and polished to make sure the uniformity and have been kept under the condition of 55% relative humidity and 23 °C for more than 24 h. The lateral and bottom surfaces of the samples were wrapped up with aluminum foil during the experiments, which guarantees the one dimensional heating.

### 2.2. Methods and Measurements

A cone calorimeter, developed by the building and fire laboratory of NIST (Gaithersburg, MD, USA) in 1982, was utilized to test the HRR and combustion product of the tested materials.

**Figure 2:**
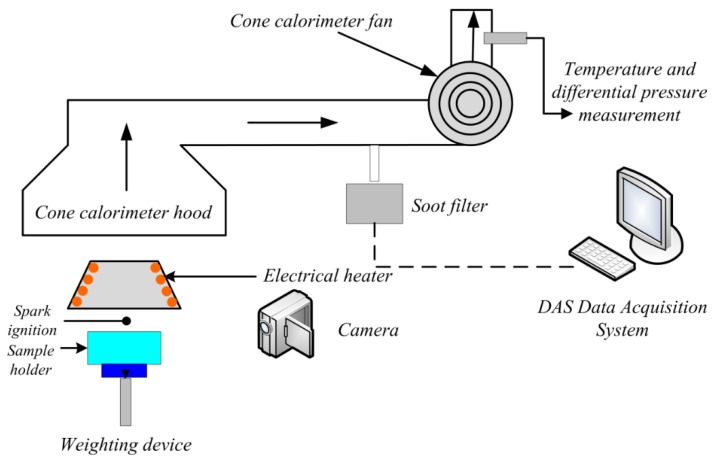
The structure of the cone calorimeter.

As shown in Figure 2, the cone calorimeter consists of a gas concentration analyzer, a combustion platform, a ventilation system, a weighting device, a gas species sampling equipment and other auxiliary devices. On the combustion platform, there are radiation heater, hood, spark ignition and circuit control system. The heating unit of heater is an electrical heating stick, the radiant heat flux ranges from 0 kW/m^2^ to 100 kW/m^2^. During the experiments, the appropriate radiance for different conditions needed to be chosen, and the materials need to be put onto the combustion platform to be ignited by the electrical spark. Fire smoke will be exhausted by the ventilation system. The gas concentration analyzer is a major element of the cone calorimeter, the gas species sampling equipment is in the ventilating tube, which determines the smoke yields of the material by measuring the shading coefficient of fire smoke. DAS (data acquisition system) records the data of oxygen analyzer, orifice meter, thermocouples and weighting device.

The fire hazards and burning behavior of the polyurethane/aluminum-polyurethane were investigated here using a Fire Testing Technology cone calorimeter, the heat flux varies from 25 kW/m^2^ to 55 kW/m^2^ with an increment of 5 kW/m^2^. Time to ignition, heat release rate, fire hazards were discussed based on their performance under various radiant heat flux, finally a comprehensive assessment of their fire hazards were performed according to the criteria proposed.

## 3. Results and Discussion

### 3.1. Time to Ignition

Thermally-thick material refers to the kind of material that the magnitude of the heat penetration $\delta =\sqrt{\gamma t_{ig}}$ is much smaller than the thickness of the material *H*. Itis usually assumed as one dimensional semi-infinite solid in thermally-thick material ignition models. With the assumption that the thermal properties of the solid are constants and with no inner heat source, the transient heat transfer equation for the solid material is:

(1) $$\frac{\partial^{2}T}{\partial x^{2}}=\frac{1}{\alpha }\frac{\partial T}{\partial t}$$

Initial condition:

(2) $$T\left(x,t\right)=T_{0},t=0$$

Under the constant external heat flux, boundary conditions are:

(3) $$-k\frac{\partial T}{\partial x}={\dot{q}}_{ext}^{\prime \prime },x=0$$

(4) $$T=T_{\infty },x=\infty$$

The temperature distribution within the solid can be expressed as follow by solving the partial differential equation:

(5) $$T=T_{0}+\frac{2{\dot{q}}_{ext}^{\prime \prime }\sqrt{\alpha t/\pi }}{k}\exp\left(\frac{-x^{2}}{4\alpha t}\right)-\frac{{\dot{q}}_{ext}^{\prime \prime }x}{k}\text{erfc}\left(\frac{x}{2\sqrt{\alpha t}}\right)$$

And then the surface temperature (*x* = 0) is derived as:

(6) $$T_{s}=T_{0}+\frac{2{\dot{q}}_{ext}^{\prime \prime }\sqrt{\alpha t/\pi }}{k}$$

If the surface temperature $T_{s}=T_{ig}$ is taken as the criterion, the relationship between ignition time and external heat flux can be obtained:

(7) $$\frac{1}{\sqrt{t_{ig}}}=\frac{1}{\sqrt{\pi }}\frac{2{\dot{q}}_{ext}^{\prime \prime }}{\sqrt{k\rho c}\left(T_{ig}-T_{0}\right)}$$

Based on the previous researches [9,10], the ignition time is influenced by the external heat flux and critical ignition temperature. In this paper, ignition time is defined as the time from the beginning of the exposure of radiation to the appearance of gas flame. Based on the model analysis above, for the thermally-thick materials, $1/\sqrt{t_{ig}}$ is proportional to the external heat flux ${\dot{q}}_{ext}^{\prime \prime }$.

The critical heat flux (CHF) is an important parameter to predict the ignition characteristic of combustibles, the value is between the minimum and maximum ignition flux. It reflects the ignition difficulty of the materials. Besides it is usually used as the criterion for ignition because of its easy observation in research especially in the testing of combustion characteristic of materials. There is also a relationship between the CHF and the external heat flux ${\dot{q}}_{ext}^{\prime \prime }$based on the previous researches [21].

(8) $${\dot{q}}_{ext}^{\prime \prime }=\frac{1}{\varepsilon }\left[h_{c}\left(T_{ig}-T_{0}\right)+\varepsilon \sigma T_{ig}^{4}\right]\equiv CHF$$

(9) $$\frac{1}{\sqrt{t_{ig}}}=\left[\frac{\varepsilon }{\sqrt{\left(\pi /4\right)k\rho c}\left(T_{ig}-T_{0}\right)}\right]\cdot {\dot{q}}_{ext}^{\prime \prime }-\left[\frac{h_{c}\left(T_{ig}-T_{0}\right)+\varepsilon \sigma T_{ig}^{4}}{\sqrt{\left(\pi /4\right)k\rho c}\left(T_{ig}-T_{0}\right)}\right]$$

The slope of the plot obtained from the equation ${t_{ig}}^{-1/2}=f\left({\dot{q}}_{ext}^{\prime \prime }\right)$ allows the computation of different thermal properties such as theoretical *CHF* by using the following equations:

(10) $$Slope=\left[\frac{\varepsilon }{\sqrt{\left(\pi /4\right)k\rho c}\left(T_{ig}-T_{0}\right)}\right]$$

(11) $$x_{\mathrm{int}ercept}=-\left[\frac{h_{c}\left(T_{ig}-T_{0}\right)+\varepsilon \sigma T_{ig}^{4}}{\sqrt{\left(\pi /4\right)k\rho c}\left(T_{ig}-T_{0}\right)}\right]$$

(12) $$CHF=-\left[\frac{x_{\mathrm{int}ercept}}{Slope}\right]$$

**Figure 3:**
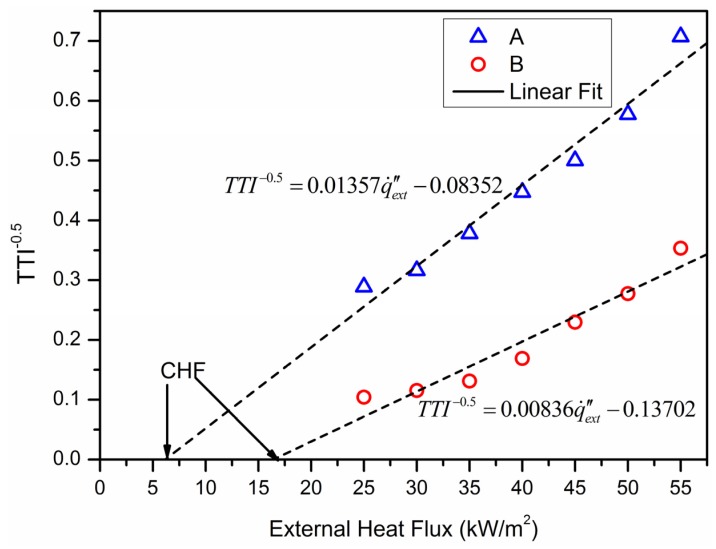
The change of ignition time with external heat flux.

By analyzing the experimental data, the relationship between $t_{ig}^{-0.5}$ and ${\dot{q}}_{ext}^{\prime \prime }$ can be illustrated in Figure 3. As it is shown, the slopes of the two straight lines by linear fitting are different, and the intercepts of the horizontal axis are critical heat fluxes for the two tests. The correlations of $t_{ig}^{-0.5}\propto {\dot{q}}_{ext}^{\prime \prime }$ for two experimental samples A and B can be written as:

(13) $$TT{I_{A}}^{-0.5}=0.01357{\dot{q}}_{ext}^{\prime \prime }-0.08352$$

(14) $$TT{I_{B}}^{-0.5}=0.00836{\dot{q}}_{ext}^{\prime \prime }-0.13702$$

Deduced from Equations (13) and (14), the CHF for material A: *CHF_A_* = 6.15 kW/m^2^, and for material B: *CHF_B_* = 16.39 kW/m^2^. It is obvious that the fire performance of the aluminum-polyurethane is much better than pure polyurethane.

### 3.2. Heat Release Rate

HRR, the most significant parameter for the fire hazard material evaluation, determines the fire growth rate. This parameter is measured by the oxygen consumption calorimeter technique. The relationship between HRR and mass loss rate (MLR) can be expressed as $HRR=\Delta h_{c}\times MLR$, where $\Delta h_{c}$ is the effective heat of combustion.

**Figure 4:**
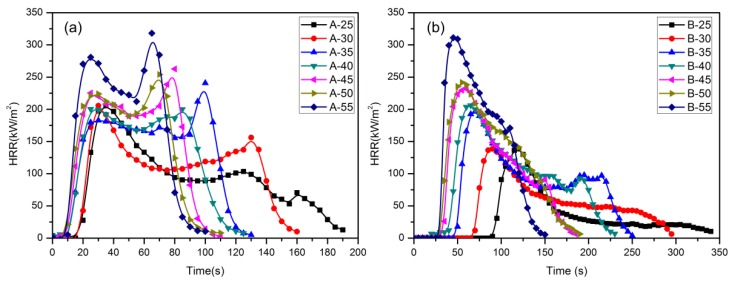
The HRR profile of material A (**a**); and material B (**b**) under different heat fluxes.

Figure 4 shows the trends of HRR for material A and B under different heat fluxes: two peak values for material A can be observed while only one for B. The HRR of material A increased very fast to approach the first peak value shortly after the piloted ignition, then reduced gradually. The reducing process maintained for a relatively long time, which was the fully burning period of the material. After that, the HRR approached the second peak value followed by a dramatic decrease due to the burn-out of the material. However, there is only one peak value for material B. The HRR decreases gradually after the peak value, which indicated that the aluminum effectively suppressed the continuous combustion of the polyurethane.

It can be seen from Figure 4a that the HRR of material A increases with heat flux, and the increasing rate also goes up with higher heat flux. The combustion becomes stronger, and the interval between two peaks becomes smaller with the increase of heat flux. The first peak of material A occurred at about 28 s with the HRR of 275 kW/m^2^, while the second peak occurred at about 68 s with the HRR of 318 kW/m^2^. The interval between two peaks is about 40 s. No distinct discrepancies of ignition times of material A can be found at different heat fluxes. In Figure 4b, the HRR curves become wider with the increase of heat flux, and the reductions of HRR indicated the radiation absorbed by materials become less, especially the feedback heat to the material itself for different heat fluxes, thus less material was brunt and less heat was released. If the circuit of feedback heat to the material is restrained, the combustion of material can be suppressed. The discrepancy of ignition time in different heat flux of material B is more obvious, especially when the heat flux is 25 kW/m^2^. The HRR of material B reduce faster than material A, which might be related to their CHF.

**Figure 5:**
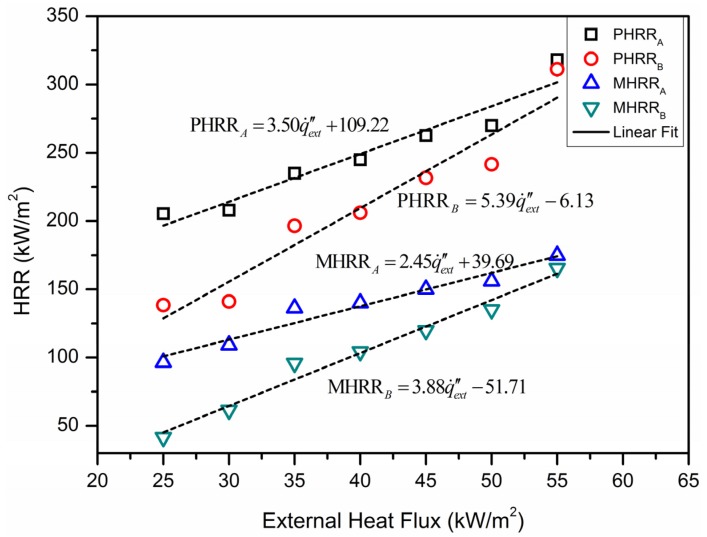
The peak and mean values of HRR for different heat fluxes.

Figure 5 shows the peak heat release rate (PHRR) and mean heat release rate (MHRR) for different heat fluxes. Basically, the magnitude of the peak and average values of HRR of material A is larger than that of material B. With the increase in heat flux, their differences decreased, especially when heat flux is 55 W/m^2^, the suppression of aluminum reduced a lot. By linear fitting, the equations of the peak and average values under external heat flux can be written in the form as:

(15) $$\text{PHRR}=a\cdot {\dot{q}}_{ext}^{\prime \prime }+b$$

(16) $$\text{MHRR}=c\cdot {\dot{q}}_{ext}^{\prime \prime }+d$$

With these equations, the peak and average values of HRR under different heat fluxes can be well predicted, and the fitted equations can be seen from Figure 5.

Based on the experimental results, the external heat flux influences the peak values of HRR and the corresponding time to reach the peak value for thermally-thick materials. With the increase in heat flux, the burning rate and the PHRR of the material increase, meanwhile the time to approach the peak decreases inversely. When the heat flux is 20 kW/m^2^, the burning rate reduced more quickly than other two heat fluxes.

There is a steady-state combustion stage for each material. The reduction of HRR becomes more slowly when the external heat flux is lower. Because of heat loss on the surface of the materials, the radiant heat has a limited influence to the combustion of the rest of the material. The material maintains the steady-state combustion mostly by the heat released from its own.

**Figure 6:**
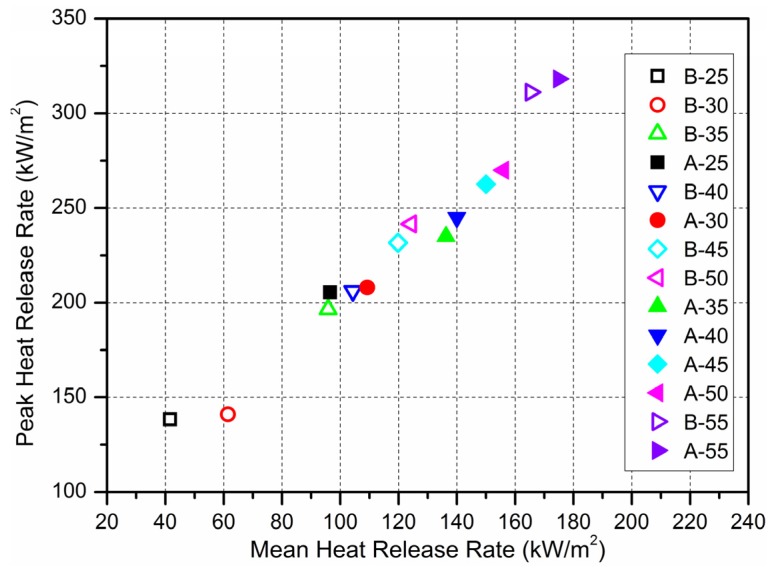
Plot of PHRR against MHRR for the material A and B tested at different external heat fluxes.

Figure 6 shows the peak values with average values of HRR of materials in 14 experimental conditions under different heat fluxes. The solid data points are for material A, and the hollow points for material B.

A linear relationship between PHRR and MHRR for the materials was found over the range of external heat fluxes used. Figure 6 shows the plot of PHRR with MHRR for the material A and B tested at heat flux varying from 25 kW/m^2^ to 55 kW/m^2^ with a step of 5 kW/m^2^, and a clear linear relationship can be found for all heat flux levels. This also indicates that the correlation between PHRR and MHRR is not dependent on the external heat flux, at least over the heat flux range studied here. With increasing heat flux, the distinction between the two materials decreases, which indicates that the effects of aluminum sheet become weaker.

**Figure 7:**
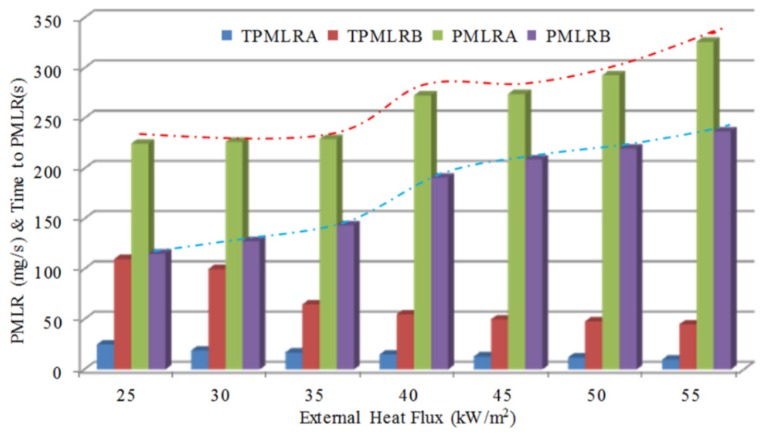
The maximum mass loss rate and the time to maximum with heat flux.

Figure 7 shows the trend of the maximum mass loss rate and the corresponding time to reach the maximum value with heat fluxes. Basically, the mass loss rates of A and B increased with increasing heat flux. The discrepancy between A and B is not obvious, which was maintained at about 90 mg/s. The time to maximum mass loss rate is inversely proportional to heat flux, especially when heat flux is 55 kW/m^2^, it needs only 10 s to approach the peak value, 326 mg/s.

### 3.3. Hazards Assessment

**Figure 8:**
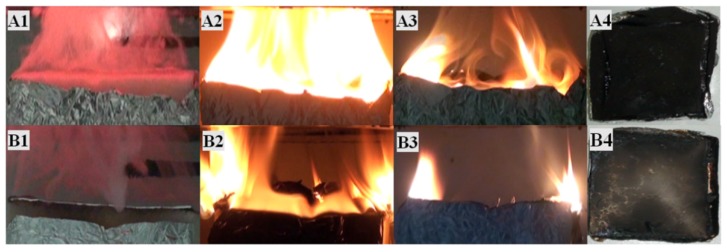
Four stages of the burning behavior of material A and B. (**A1**) Ignition; (**A2**) Flashover; (**A3**) Decay; (**A4**) Extinction; (**B1**) Ignition; (**B2**) Flashover; (**B3**) Decay; (**B4**) Extinction.

A series of experiment photos for material A and B are shown in Figure 8. Four phases of combustion can be identified. Grey combustible gas was volatilized from the materials under heat flux and the material expanded gradually during the pyrolysis, which is rather obvious in phase A1/B1. As the combustible gas above the surface of the solid material was mixed with the air, the flame emerged once the burning condition was met. In the second phase A2/B2 after ignition, the combustion gets more fiercely, and material A was wholly covered by the flame. While with the impediment of aluminum sheet, the flame above material B was unstable and smaller than material A.

In the phase A3/B3, A charring layer was formed on the top surface of both material A and B, which affected the release of volatile gas. However, its influence is weaker than the influences of aluminum sheet. After extinction, material A is totally burned out and material B was left with a curved aluminum sheet. It is indicated from observation that the performance of fire resistance of material B is much better than that of material A.

The parameter *x* was proposed by Petrella [19,28] for studying the contribution of the materials to flashover. The parameter, flashover propensity, *x* (kW·m^−2^·s^−1^) is defined as:

(17) $$x=\frac{PHRR}{TTI}$$

in which, TTI refers to time to ignition.

Table 1 lists the standardization of parameter *x*, and the values of *x* listed in Table 2 then can be calculated based on Equation (17) and the experimental data. With “intermediate” means the hazards is under control, “High” means attention care should be taken when using that material, and “very high” means extra care is needed.

Also Table 2 presents the classification of fire hazard levels for materials under different experimental conditions. The fire hazards of material is evaluated based on the flashover and the total heat release rate. Although the value *y* is about 18 due to the small size of experimental samples, it is of intermediate risk to heat contribution overall. The average value of material *x* is above 10, indicating that it is seriously hazardous. When heat flux is 25–40 kW/m^2^, the average *x* is smaller than 10, which is of intermediate risk to flashover. Again, it indicates that the quality of fire resistance of material B is much better than that of material A.

Previous researches have rarely taken the fire risk of building thermal-insulation material into consideration, especially the smoke hazard of it. The study of fire hazards above analyzed the combustible characteristic and heat release rate. Later, more focus will be put on the study of toxic gas yielded from the combustible materials.

### 3.4. Toxic Fire Hazards

**Figure 9:**
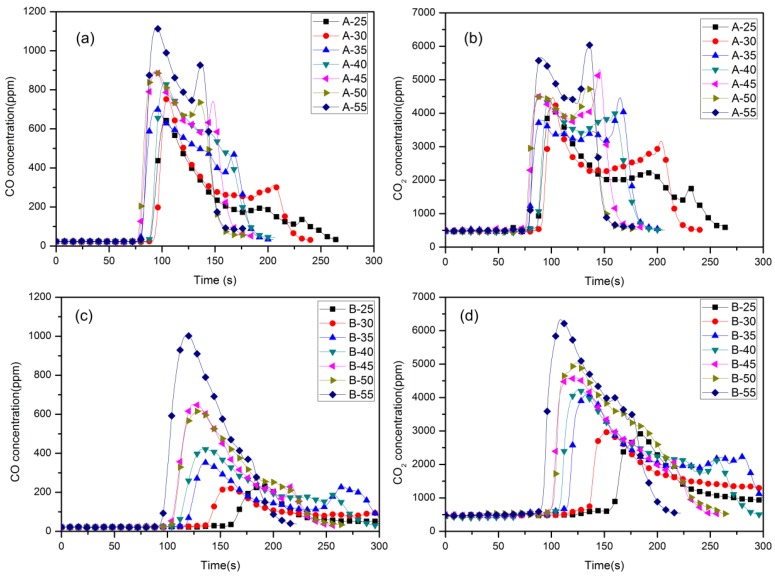
CO and CO_2_ concentration of material in different heat fluxes.

CO is the most toxic component in fire gases, preventing O_2_ transportation by the formation of carboxyhaemoglobin. What can be seen in Figure 9 is the CO and CO_2_ concentration generated from material A and B in different heat fluxes. It can be observed from Figure 9 that the CO yields of material A varied from 645 ppm to 1117 ppm and 224 ppm to 1005 ppm of material B under different radiant heat fluxes, and the CO_2_ yields of material A varied from 4116 ppm to 6038 ppm and 2916 ppm to 6331 ppm of material B under different radiant heat fluxes. Note that most of the material loss was PU, not the aluminum sheet.

Overall, the toxic gas concentration generated from material A is much higher than material B, especially when the heat flux is below 50 kW/m^2^. For example, the CO concentration is 895 ppm of material A when heat flux is 45 kW/m^2^, which is much higher than 650 ppm of material B.

**Figure 10:**
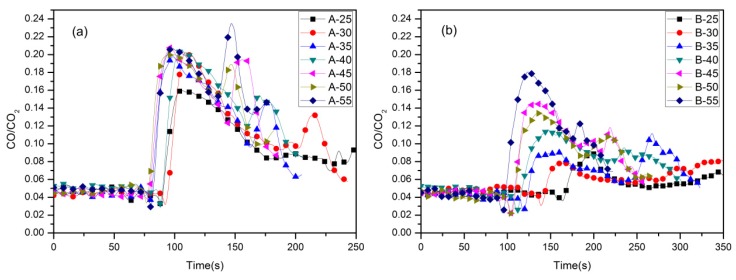
CO/CO_2_ ratio in relation to heat flux. (**a**) aluminum-polyurethane; (**b**) pure polyurethane.

The ratio of CO/CO_2_ is also a very important factor in evaluating the fire hazards of fire gas yields, the bigger CO/CO_2_ is, the higher CO concentration in the yields of burning and the more serious of fire hazard will be. Shown in Figure 10, the ratio of CO/CO_2_ for material A is much higher than material B after ignition and it rises to a peak value during the burning process, the maximum of which is 0.23, however the ratio of CO/CO_2_ for material B increases slowly, which indicates the fire hazards of material B is smaller than A.

**Figure 11:**
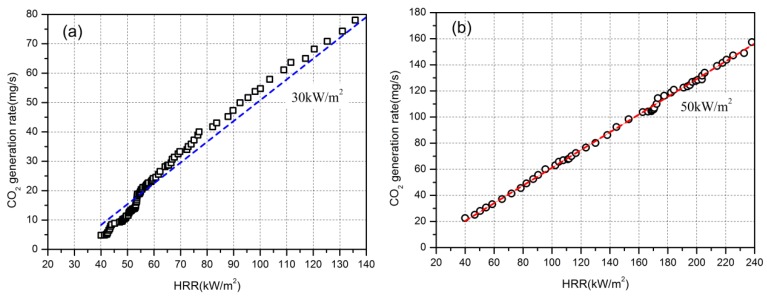
Generation rate of CO_2_ for material B with HRR in the heat flux of 30kW/m^2^ (**a**); and 50kW/m^2^ (**b**).

The CO_2_ yielding rate is another important parameter for fire hazards assessment. From Figure 11, it can be seen that there exists a liner relationship between the CO_2_ yielding rate and the HRR. With liner correlation equations shown below, the CO_2_ yielding rate with different HRR can be calculated.

(18) $${\overset{B-30}{GR}}_{CO_{2}}=0.71\times HRR-20$$

(19) $${\overset{B-50}{GR}}_{CO_{2}}=0.68\times HRR-7$$

From equations above, the CO_2_ yielding rate can be well predicted based on the heat release rate, for example when the heat flux is 50 kW/m^2^ and HRR is 150 kW/m^2^, the CO_2_ yielding rate can be calculated to be around 95 mg/s.

To evaluate the fire hazard of major gas species, previous researchers has developed the N-gas model [23,24], this model assumed that the fire hazards of all yields were mostly caused by several major gases. In the N-gas model, *N* represents the observed toxic gas, then the value of all the toxic gas will be accumulated.

(20) $$N_{Gas}=\frac{m[CO]}{[CO_{2}]-b}+\frac{21-[O_{2}]}{21-LC_{50}(O_{2})}+\frac{\left[HCN\right]}{LC_{50}(HCN)}+\frac{0.4[NO_{2}]}{LC_{50}(NO_{2})}+\frac{\left[HCN\right]}{LC_{50}(HCN)}+\frac{\left[HBr\right]}{LC_{50}(HBr)}$$

*m* and *b* were determined by the experiments and were −18 and 122000 respectively when CO_2_ concentration is below 5%, and is 23 and −386000 respectively when CO_2_ concentration is above 5%. The criterion of N-gas model is:

1. When *N* ≈ 1, parts of experimental animals died;
2. When *N* < 0.8, no experimental animals died;
3. When *N* > 1.3, all experimental animals died.

FED method has combined LC_50_ standard and N-gas model, which has taken the time accumulation effect into consideration, and it assumed that the hazard of different gases is linear, the equation is [28]:

(21) $$FED=\sum_{i}\frac{\int_{0}^{t}C_{i}dt}{LC_{50}(i)t}$$

By following Babrauskas, FED can be estimated by using only the *pk*[*CO*]:

(22) $$FED=\frac{pk[CO]}{5000}$$

To better study the hazards of toxic gases during the whole combustion period, a peak width time was proposed, which is the sum of the time when the concentration of toxic gas in the fire smoke is higher than the median value, by which the lasting time of high concentration gas in the experiments can be evaluated to measure the hazards of smoke.

**Figure 12:**
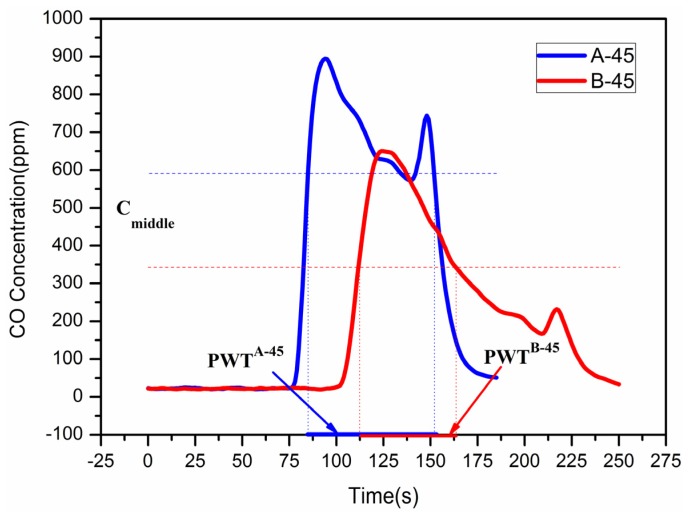
CO concentration generated by material A and B when heat flux is 45 kW/m^2^.

Figure 12 shows that the CO concentration generated by material A and B when heat flux is 45 kW/m^2^, the horizontal line is the position of the median of numerical integration. The intersection point on the left side of the horizontal line and the experimental data is the beginning of the peak width time. The ending of the peak width time is the right side of the horizontal line and the experimental data crossed. The peak width time is 67 s for material A and 53 s for material B when heat flux is 45 kW/m^2^.

The peak width time is the major factor reflecting the lasting time of gas hazard in the experiments. However, these factors cannot show the influence of the toxic gases comprehensively. In this paper, based on the comprehensive influence of time and gas concentration, the hazard factor *HI* (Hazard Index) was introduced as a new factor to develop the hazard model. The calculating method is described below.

(1) The total amount toxic gases by numerical integration
  (23) $$V=\int_{t_{begin}}^{t_{end}}C(t)dt,t_{beign}\leq t\leq t_{end}$$
  $t_{beign}$,$t_{end}$ is the time when CO concentration in the testing point is 10% higher or lower than a constant value respectively. *C* (*t*) is the concentrations of toxic gas at different time. *V* is the whole volume of the toxic gas.
(2) The integral median concentration by median principle:
  (24) $$C_{middle}=\frac{V}{t_{end}-t_{begin}}$$
  $C_{middle}$ is thus the calculated integral median concentration of toxic gas.
(3) Peak width time (*PWT*)
  (25) $$PWT=\sum_{t_{begin}}^{t_{end}}t_{select},C\left(t_{select}\right)-C_{middle}>0$$
  $t_{select}$ is the time when the CO concentration is above the integral mid-value, *PWT* is the calculated peak width time.
(4) The hazard index *HI*:
  (26) $$HI=\frac{C_{middle}\cdot PWT}{\beta \Phi_{\mathrm{de}ath}^{CO}}$$
  $\Phi_{\mathrm{de}ath}^{CO}$ is the extremum amount of CO of death, and is taken as 12800×60ppm⋅s [28]. β is the undetermined coefficient which is 0.2578 in the experiments.

**Figure 13:**
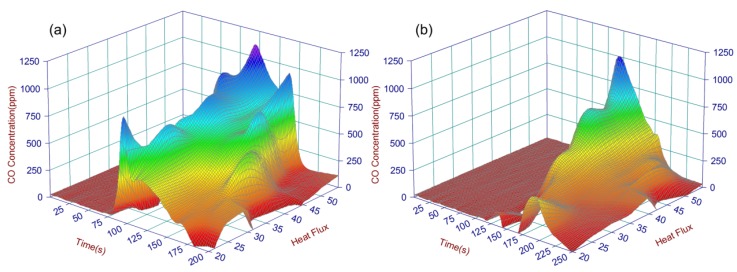
3D illustrations for CO concentrations of material A (**a**); and B (**b**) in different heat flux.

A lot of gas species tests have been detected, a series of data points {*C_k_*(*t_j_*,*h_i_*)} has been received (*C* is the species concentration in time, *h* is heat flux) . In this paper, the major toxic gas CO is analyzed. Because of the difference of the testing points, interpolation method is applied to provide the data needed to avoid big deviation from the fitting results below. After the interpolation of experimental data, equidistant plane grid points of two dimensions have been formed, and it is shown that the result is not confusing the original information. Lagrange Interpolation formula was used here to avoid the spread and amplification of error caused by high degree interpolation. Figure 13 reflects the distribution of CO concentration generated from the combustion of material A and B. It is obvious that there are more peak points for A, and all of them are larger than B’s, which indicated the fire hazard of B is lighter than A’s, then the hazard index can be used to analyze the fire hazard of the materials.

**Figure 14:**
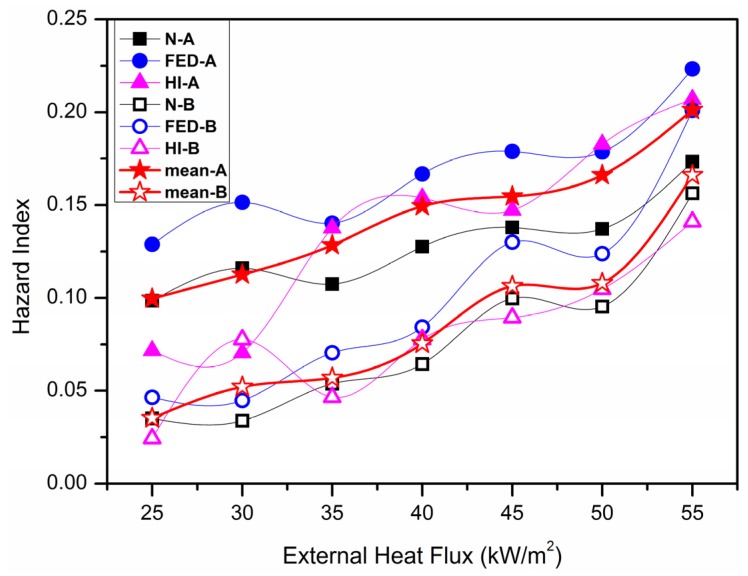
Fire hazard models for material A and B.

Figure 14 is the contraction for fire hazard models of material A and B calculated by *HI* model combined with the peak width time to the classic N-gas model and FED model. It can be seen the calculated results of the three analyzing models seemed to be similar to one another, as the FED model is modified based on the N-gas model, so the results from these two models are close. To better analyze the fire hazard index of material, *HNF* is defined as follow,

(27) $$HNF=\sum_{1}^{n}w_{n}\cdot Q_{n}$$

where *w* is the weighted coefficient, and *Q* is the hazard model.

By calculating and amending the arithmetic average data got from the three models, three weighted coefficient of N-gas model, FED model and HI hazard model can be deduced.

(28) $$HNF=w_{1}N_{Gas}+w_{2}FED+w_{3}HI$$

**Figure 15:**
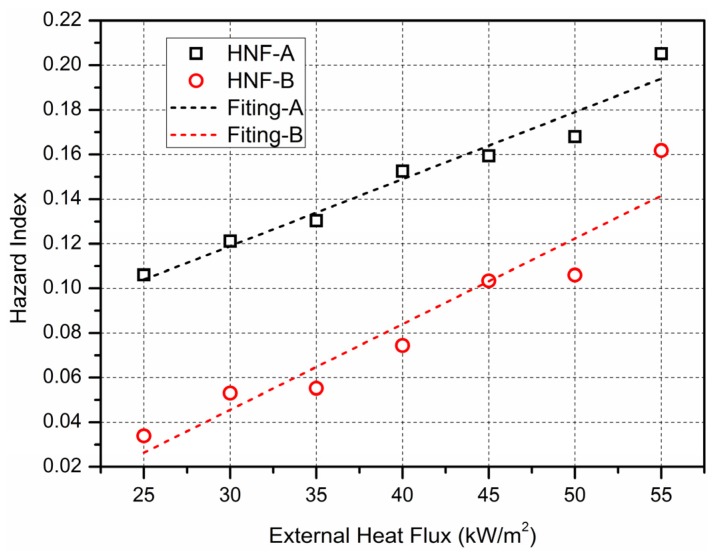
HNF model of fire hazard for material A and B.

Figure 15 is HNF model of fire hazard for material A and B, which is calculated from the experimental data, from which it can be seen that the HNF value of material increases with the heat flux, and between them a linear relationship is maintained. It also can be seen that the value of HNF for material A is bigger than B. The value of HNF for B is smaller than 0.074 when heat flux is less than 40 kW/m^2^, which is lower than the value of HNF 0.106 for material A when heat flux is less than 25 kW/m^2^. From the analysis above one can infer that, HNF model can better evaluate and predict the hazard of toxic gas generated from combustion, which is of vital importance for the study of fire hazard in building insulation material.

## 4. Conclusions

In this paper, the fire hazards for insulation materials used in diesel locomotive under different radiant heat flux have been studied. By studying the relationship between ignition time, external heat flux and the critical heat flux, the critical heat flux of pure-polyurethane is found to be around 6.15 kW/m^2^, and aluminum-polyurethane is 16.39 kW/m^2^. The equation for the maximum and average HRR in different heat fluxes and the relationship of the maximum mass loss rate with the time approaching the maximum value have also been discussed. The fire hazard of material has been evaluated from two perspectives, flashover and the whole HRR, the fire hazard has been classified under different experimental conditions. It is found the fire hazard of aluminum-polyurethane is smaller than that of pure polyurethane. An index of Peak Width Time was proposed to better analyze the hazard of solid material burning. A new hazard index HI was proposed, which was the product of PWT and median concentration. It is found in the experiments that the calculated HI is consistent with the existed N-gas and FED models for evaluating the fire gas hazard. The integrated model HNF was proposed as well by applying two dimension interpolations and weighted average calculation to evaluate the fire hazards of material.

## Figures and Tables

**Table 1:** Two parameters for predicting fire hazard.

Values	*x* (Flashover)
0.1–1.0	Low risk to flashover LRF
1.0–10	Intermediate risk to flashover IRF
10–100	High risk to flashover HRF
100–1000	Very high risk to flashover VHRF

**Table 2:** Classification of fire hazard grade for material in different experimental conditions.

Test	Heat Flux (kW·m^−2^)	Parameters	
		*x = PHRR/TTI* (kW·m^−2^·s^−1^)	Risk Classification
A-25	25	17.12	High
A-30	30	20.80	High
A-35	35	33.57	High
A-40	40	49.00	High
A-45	45	65.66	High
A-50	50	90.00	High
A-55	55	159.09	Very high
B-25	25	1.50	Intermediate
B-30	30	1.88	Intermediate
B-35	35	3.39	Intermediate
B-40	40	5.89	Intermediate
B-45	45	12.19	High
B-50	50	18.59	High
B-55	55	38.90	High

## Abbreviations

The following abbreviations are used in this manuscript:

### *Nomenclature*

*A*: Polyurethane
*B*: Aluminum-polyurethane
*C* (*t*): Concentrations of toxic gas at different time
*CHF*: Critical heat flux (kW·m^−2^)
*HRR*: Heat release rate (kW·m^−2^)
*HI*: Hazard index
$h_{c}$: Convective heat transfer coefficient (W·m^−2^·K^−1^)
*k*: Thermal conductivity (kW·m^−1^·K^−1^)
*MLR*: Mass loss rate (g·s^−1^)
*m*: Mass (g)
*PWT*: Peak width time (s)
*Q*: The hazard model
${\dot{q}}_{ext}^{\prime \prime }$: External heat flux (kW·m^−2^)
*THR*: Total heat release (MJ·m^−2^)
*TTI*: Time to ignition (s)
$T_{ig}$: Ignition temperature (K)
$T_{0}$: Ambient temperature (K)
*t*: Time (s)
$t_{ig}$: Time to ignition (s)
*V*: The volume of the toxic gas

### *Greek* *symbols*

α: Heat transfer coefficient (m^2^·W^−1^·s^−1^)
β: The undetermined coefficient according to experiments
γ: Heat penetration coefficient
*w*: Weighted coefficient
δ: The thickness of heat penetration (m)
ε: Emissivity
*σ*: Stefan-Boltzmann constant (W·m^−2^·K^−4^)
$\Phi_{\mathrm{de}ath}^{CO}$: The CO limit of death
*ρ*: Density (kg·m^−3^)
$\Delta h_{c}$: Effective combustion heat (MJ/kg)
